# Oxaliplatin Neurotoxicity Involves Peroxisome Alterations. PPARγ Agonism as Preventive Pharmacological Approach

**DOI:** 10.1371/journal.pone.0102758

**Published:** 2014-07-18

**Authors:** Matteo Zanardelli, Laura Micheli, Lorenzo Cinci, Paola Failli, Carla Ghelardini, Lorenzo Di Cesare Mannelli

**Affiliations:** Dept. of Neuroscience, Psychology, Drug Research and Child Health - Neurofarba - Pharmacology and Toxicology Section, University of Florence, Florence, Italy; Nazarbayev University, Kazakhstan

## Abstract

The development of neuropathic syndromes is an important, dose limiting side effect of anticancer agents like platinum derivates, taxanes and vinca alkaloids. The causes of neurotoxicity are still unclear but the impairment of the oxidative equilibrium is strictly related to pain. Two intracellular organelles, mitochondria and peroxisomes cooperate to the maintaining of the redox cellular state. Whereas a relationship between chemotherapy-dependent mitochondrial alteration and neuropathy has been established, the role of peroxisome is poor explored. In order to study the mechanisms of oxaliplatin-induced neurotoxicity, peroxisomal involvement was evaluated in vitro and in vivo. In primary rat astrocyte cell culture, oxaliplatin (10 µM for 48 h or 1 µM for 5 days) increased the number of peroxisomes, nevertheless expression and functionality of catalase, the most important antioxidant defense enzyme in mammalian peroxisomes, were significantly reduced. Five day incubation with the selective Peroxisome Proliferator Activated Receptor-γ (PPAR-γ) antagonist G3335 (30 µM) induced a similar peroxisomal impairment suggesting a relationship between PPARγ signaling and oxaliplatin neurotoxicity. The PPARγ agonist rosiglitazone (10 µM) reduced the harmful effects induced both by G3335 and oxaliplatin. In vivo, in a rat model of oxaliplatin induced neuropathy, a repeated treatment with rosiglitazone (3 and 10 mg kg^−1^ per os) significantly reduced neuropathic pain evoked by noxious (Paw pressure test) and non-noxious (Cold plate test) stimuli. The behavioral effect paralleled with the prevention of catalase impairment induced by oxaliplatin in dorsal root ganglia. In the spinal cord, catalase protection was showed by the lower rosiglitazone dosage without effect on the astrocyte density increase induced by oxaliplatin. Rosiglitazone did not alter the oxaliplatin-induced mortality of the human colon cancer cell line HT-29. These results highlight the role of peroxisomes in oxaliplatin-dependent nervous damage and suggest PPARγ stimulation as a candidate to counteract oxaliplatin neurotoxicity.

## Introduction

Oxaliplatin is a chemotherapeutic compound widely used for treating colorectal cancer [1]. The development of sensory neuropathy is the most important, dose-limiting side effect. Platinum-induced peripheral neuropathy is characterized by distal paresthesias and mild muscle contractions for at least 80% of oncologic patients after few hours to days from the first oxaliplatin infusion [2], [3]. Moreover, oxaliplatin repeated treatment induces severe peripheral neuropathy that can affect approximately 50% of the patients receiving cumulative doses higher than 1000 mg/m^2^ [4], [5]. Anti-hyperalgesic compounds currently used to treat chemotherapy-induced pain, like antiepileptics or antidepressant, are weakly effective [6]. The therapeutic failure reflects the lack of knowledge about the molecular bases of neuropathies. In a rat model of oxaliplatin-induced neuropathy we previously identified oxidative stress as a main biomolecular dysfunction showing a relationship between oxidative damage of the nervous system and pain [7]. The “oxidative hypothesis” was confirmed in primary cultures of astrocytes [8], a glial cell type activated in vivo by oxaliplatin treatment [9]. Since oxaliplatin does not possess direct oxidative properties [8], redox unbalance seems due to a cell-mediated effect able to alter the oxidative machinery.

After oxaliplatin treatment, mitochondria are modified in morphology and impaired in function [10]. Less inquired is the role of the other intracellular organelle strongly implied in redox processes: the peroxisome. Peroxisomes are the last among the subcellular organelles to be identified [11]. The discovery of the co-localization of catalase with H_2_O_2_-generating oxidases in peroxisomes was the first indication of their involvement in the metabolism of oxygen metabolites [11]. The high peroxisomal consumption of O_2_, the demonstration of the production of H_2_O_2_, O_2_^−^, ·OH, and more recently of ·NO [11]–[14], as well as the discovery of several ROS metabolizing enzymes in peroxisomes has supported the notion that these ubiquitous organelles play a key role in both the production and scavenging of ROS in the cell [15]. In the nervous system, the functional relevance of these organelles is dramatically highlighted by peroxisomal disorders. Severe demyelination, axonal degeneration and neuroinflammation are induced by genetic deficit of peroxisome [16]–[19]. Moreover, peroxisomes were recently involved in the development and progression of specific degenerative diseases [18], [20]–[22].

In mouse liver was originally cloned a nuclear receptor subfamily of ligand-activated transcription factors, the Peroxisome Proliferator-Activated Receptors (PPARs) [23]. PPARs may activate genes with a PPAR response element (PPRE) in their promoter regions [24]. Girnun et al. [25] highlighted that PPARγ stimulation increases the expression and activity of catalase, a heme-containing peroxisomal enzyme that breaks down hydrogen peroxide to water and oxygen [26], [27]. Recently, agonists of the γ subtype of PPARs received considerable attention as potential therapeutic agents for a wide range of neurological diseases, including neurodegenerative diseases, traumatic injuries, stroke and demyelinating diseases [28]–[38].

Aimed to characterize the oxaliplatin neurotoxicity, we studied the peroxisome-related signal in vitro, in astrocyte cell culture, and in vivo in a rat model. Peroxisome stimulation by the PPARγ agonist rosiglitazone was analyzed to individuate new possible pharmacological approaches to control oxaliplatin-induced neuropathy.

## Materials and Methods

### Astrocyte cultures

Primary cultures of astrocytes were obtained according to the method described by McCarthy and de Vellis [39]. Briefly, the cerebral cortex of newborn (P1–P3) Sprague–Dawley rats (Harlan, Italy) was dissociated in Hanks’ balanced salt solution containing 0.5% trypsin/EDTA and 1% DNase (Sigma-Aldrich, Milan, Italy) for 30 min at 37°C. The suspension was mechanically homogenized and filtered. Cells were plated in high-glucose Dulbecco’s Modified Eagle’s Medium (DMEM) with 10% fetal bovine serum (FBS, Gibco, Invitrogen, Milan, Italy). Confluent primary glial cultures were used to isolate astrocytes, removing microglia and oligodendrocytes by shaking. The purity of astrocyte cultures was determined immunocytochemically by staining for GFAP (Dako, Glostrup, Denmark). Cells were fixed in 4% paraformaldehyde, then incubated with the antibody (1∶200), and visualized using Alexa Fluor-conjugated secondary antibody (Life Technologies, Monza, Italy). Nuclei were stained with 4,6-diamidino-2-phenylindole dihydrochloride. 90% of cells in astrocyte cultures were GFAP-positive. Experiments were performed 21 days after cell isolation. Formal approval to conduct the experiments described was obtained from the Animal Subjects Review Board of the University of Florence. The ethics policy of the University of Florence complies with the Guide for the Care and Use of Laboratory Animals of the U.S. National Institutes of Health (NIH Publication No. 85-23, revised 1996; University of Florence Assurance No. A5278-01).

The human colon cancer cell line HT-29 was obtained from American Type Culture Collection (Rockville, MD). HT-29 were cultured in DMEM high glucose with 20% FBS in 5% CO_2_ atmosphere at 37°C. Media contained 2 mM L-glutamine, 1% essential aminoacid mix, 100 IU ml^−1^ penicillin and 100 µg ml^−1^ streptomycin (Sigma, Milan, Italy).

### Cell treatments

On day 21, astrocytes were plated in 12-wells cell culture (2·10^5^/well; Corning, Tewksbury MA, USA), or on polylysine-coated slides (5·10^4^/well) and experiments were performed after 48 h. Cells were treated with 10-and 1 µM oxaliplatin (Sequoia Research Products, Pangbourne, UK) for 2 or 5 days respectively. Rosiglitazone (10 µM; Sequoia Research Products, Pangbourne, UK) and 30 µM G3335 (BioVision Incorporated, Milpitas, CA, USA) were used in the presence or absence of oxaliplatin for 2 or 5 days. The chosen concentrations are in accord with previous published data [8], [40], [41] and, as regards oxaliplatin, with plasmatic concentration of treated rats.

HT-29 cells were plated in 96-wells cell culture (1·10^4^/well) and, 48 h after, treated as described above.

### Cell viability assay

HT-29 cell viability was evaluated by the reduction of 3-(4,5- dimethylthiozol-2-yl)-2,5-diphenyltetrazolium bromide (MTT) as an index of mitochondrial compartment functionality. Cells were plated into 96-well cell culture plates, and treated after 48 h. Oxaliplatin, at various concentrations, was incubated in DMEM in the presence of 10 µM rosiglitazone for 48 h and 5 days. After extensive washing, 1 mg/ml MTT was added into each well and incubated for 30 minutes at 37°C. After washing, the formazan crystals were dissolved in 150 µl dimethyl sulfoxide. The absorbance was measured at 550 nm. Experiments were performed in quadruplicate on at least three different cell batches.

### Animals

For all the experiments described below, male Sprague-Dawley rats (Harlan, Varese, Italy) weighing approximately 200 to 250 g at the beginning of the experimental procedure were used. Animals were housed in CeSAL (Centro Stabulazione Animali da Laboratorio, University of Florence) and used at least 1 week after their arrival. Four rats were housed per cage (size 26×41 cm); animals were fed with standard laboratory diet and tap water ad libitum, and kept at 23±1°C with a 12 hour light/dark cycle, light at 7 a.m. All animal manipulations were carried out according to the European Community guidelines for animal care (DL 116/92, application of the European Communities Council Directive of 24 November 1986 (86/609/EEC). The ethical policy of the University of Florence complies with the Guide for the Care and Use of Laboratory Animals of the US National Institutes of Health (NIH Publication No. 85-23, revised 1996; University of Florence assurance number: A5278-01). Formal approval to conduct the experiments described was obtained from the Animal Subjects Review Board of the University of Florence. All efforts were made to minimize animal suffering and to reduce the number of animals used.

### Oxaliplatin model and pharmacological treatments

Oxaliplatin neuropathy was induced as described by Cavaletti et al. [42]. Rats were treated with 2.4 mg kg^−1^ oxaliplatin, administered intraperitoneally (i.p.) for 5 consecutive days every week for 3 weeks (15 i.p. injections). Oxaliplatin was dissolved in 5% glucose solution. Rosiglitazone at the doses of 3 and 10 mg kg^−1^ was suspended in 1% carboxymethylcellulose sodium salt (CMC) and administered per os (p.o.) daily starting from the first day of oxaliplatin administration up to day 20. Control animals received an equivalent volume of vehicles: i.p. glucose or p.o. CMC (vehicle). Behavioral, morphological and biochemical tests were performed on day 21, 24 hours after last treatments. On day 21, organic platinum plasmatic levels evaluated by Inductively Coupled Mass Spectrometry (ICP-MS) were 3.573±0.271 µg/mL (corresponding to oxaliplatin 7.274±0.552 µg/mL or 18.3 µM).

### Paw pressure test

The nociceptive threshold in the rat was determined with an analgesimeter (Ugo Basile, Varese, Italy), according to the method described by Leighton et al. [43]. Briefly, a constantly increasing pressure was applied to a small area of the dorsal surface of the hind paw using a blunt conical probe by a mechanical device. Mechanical pressure was increased until vocalization or a withdrawal reflex occurred while rats were lightly restrained. Vocalization or withdrawal reflex thresholds were expressed in grams. Rats scoring below 40 g or over 75 g during the test before drug administration were rejected (25%). For analgesia measures, mechanical pressure application was stopped at 120 g. Rats were randomly assigned to each experimental group and individually habituated to handling before testing.

### Cold Plate Test

The animals were placed in a stainless box (12 cm×20 cm×10 cm) with a cold plate as floor. The temperature of the cold plate was kept constant at 4°C±1°C. Pain-related behaviors (i.e. lifting and licking of the hind paw) were observed and the time (s) of the first sign was recorded. The cut-off time of the latency of paw lifting or licking was set at 60 s.

### Rota-Rod Test

The Rota-rod apparatus (Ugo Basile, Varese, Italy) consisted of a base platform and a rotating rod with a diameter of 6 cm and a non-slippery surface. The rod was placed at a height of 25 cm from the base. The rod, 36 cm in length, was divided into 4 equal sections by 5 disks. Thus, up to 4 rats were tested simultaneously on the apparatus, with a rod-rotating speed of 10 r.p.m. The integrity of motor coordination was assessed on the basis of the time the animals kept their balance on the rotating rod up to a maximum of 10 min (600 s). After a maximum of 6 falls from the rod, the test was suspended and the time was recorded.

### Tissue collection

On day 21, at the end of the behavioral test session, animals were sacrificed by decapitation. L4-L5 dorsal root ganglia (DRG) were dissected and frozen using liquid nitrogen. L4/L5 segments of the spinal cord were exposed from the lumbovertebral column via laminectomy and identified by tracing the dorsal roots from their respective DRG. After dissection, this lumbar portion was frozen using liquid nitrogen or fixed by immersion in 4% neutral buffered formalin. Blood was collected in heparin-treated tubes and plasma fraction was isolated by centrifugation.

### Catalase immunoreaction in astrocyte cell culture

Cytological specimens were fixed in 4% paraformaldehyde for 10 minutes and then washed in PBS. For immunolabeling, slides were treated with 0.3% H_2_O_2_ (v/v) in water to quench endogenous peroxidase and then pre- incubated for 15 minutes in Ultra V Block (Thermo Scientific, Rancom Cheshire, UK). Successively, the slides were incubated with rabbit polyclonal anti-Catalase antiserum at final dilution of 1∶100 (Novus Biological, Littelton, CO, USA). Immuno-reaction was revealed by biotin-conjugated goat anti-rabbit IgG to a final dilution of 1∶200 (Dako, Glostrup, Denmark) followed by incubation with Streptavidin Peroxidase Complex (Thermo Scientific, Rancom Cheshire, UK). The morphology, size and cellular localization allowed us to identify the stained bodies as peroxisomes.

Negative controls were carried out by omitting the primary or the secondary antibodies. Staining was performed in a single session, to minimize artifactual differences in the staining. Numerous (>10) photomicrographs of cells were randomly taken using a digital photomicroscopy apparatus with a 40× objective. Each microscopic field corresponds to a test area of 38,700 µm^2^. On the digitized images peroxisomes were counted in every single cell and cell area was also measured Measurements were carried out using ImageJ 1.33 free-share image analysis software (ImageJ, NIH, Bethesda, Maryland, USA). The results were expressed as number of peroxisomes/µm^2^. On the digitized images, measurements of optical density of catalase-immunostained peroxisomes were carried out after determining the appropriate threshold to include only immune-reactive peroxisomes. The results were expressed as mean of optical density/number of peroxisomes. After determination of number and optical density of peroxisomes, the slides were counterstained with hematoxylin to highlight the nuclei.

### Catalase activity

Enzymatic activity was measured both in astrocyte culture and in nervous tissue. After incubation, cells were washed once with PBS and scraped with PBS on ice. Cells were then collected, subjected to a freeze–thaw cycle and centrifuged (13,000×g for 10 min at 4°C). DRG and spinal cord were homogenated in PBS. The suspension was sonicated on ice using three 10 s bursts at high intensity with a 10 s cooling period between each burst and then centrifuged (13,000×g for 15 minutes at 4°C). Catalase activity was measured in the supernatant by Amplex Red Catalase Assay Kit (Invitrogen, Monza, Italy) following the manufacturer’s instructions.

Protein concentration was quantified by bicinchoninic acid assay (Sigma–Aldrich, Milan, Italy). Catalase activity for each sample was normalized to protein concentration. Control conditions in the absence of treatment were set as 100%.

### Lipid peroxidation

Thiobarbituric acid reactive substances (TBARS) were quantified in spinal cord tissue homogenate as described previously [7]. Tissue homogenate (1.5 mg) was added to 4 mL reaction mixture consisting of 36 mM thiobarbituric acid (Sigma-Aldrich, Milan, Italy) solubilized in 10% CH_3_COOH, 0.2% SDS, pH was adjusted to 4.0 with NaOH. The mixture was heated for 60 minutes at 100°C and the reaction was stopped by placing the vials in ice bath for 10 minutes. After centrifugation (at 1.600 g at 4°C for 10 minutes) the absorbance of the supernatant was measured at 532 nm (Perkin-Elmer spectrometer, Monza, Italy) and TBARS were quantified in µmoles/milligram of total protein using 1,1,3,3-tetramethoxypropane as standard. Protein homogenate concentration was measured by bicinchoninc acid (BCA; Sigma-Aldrich, Milan, Italy) assay.

### Western blotting analysis

After incubation, astrocyte cell cultures were washed once with PBS and scraped on ice with lysis buffer containing 50 mM Tris-HCl pH 8.0, 150 mM NaCl, 1 mM EDTA, 0.5% Triton X-100, Complete Protease Inhibitor (Roche, Milan, Italy). Cells were then collected, subjected to a freeze–thaw cycle and centrifuged at 13,000×g for 10 min at 4°C. Nervous tissue (DRG and spinal cord) from treated animals was homogenized in the lysis buffer described before. The suspension was sonicated on ice using three 10 s bursts at high intensity with a 10 s cooling period between each burst and then centrifuged (13,000×g for 15 minutes at 4°C). Protein concentration was quantified by bicinchoninic acid assay. Forty µg of each sample were resolved with 10% SDS-PAGE before electrophoretic transfer onto nitrocellulose membranes (Biorad, Milan, Italy). Membranes were blocked with 5% nonfat dry milk in PBS containing 0.1% Tween 20 (PBST) and then probed overnight at 4°C with primary antibody specific versus catalase (1∶1000; 60 kDa; Novus Biological, Littleton, CO, USA), GAPDH (1∶1000; 38 kDa; Cell Signaling, Boston, MA, USA). Membranes were then incubated for 1 hour in PBST containing the appropriate horseradish peroxidase-conjugated secondary antibody (1∶5000; Cell Signalling, USA). ECL (Enhanced chemiluminescence Pierce, Rockford, IL, USA) was used to visualize the peroxidase-coated bands. Densitometric analysis was performed using the “ImageJ” analysis software (ImageJ, NIH, Bethesda, Maryland, USA) and results were normalized to GAPDH immunoreactivity as internal control. Values are reported as percentages in comparison to control which was arbitrarily fixed at 100%.

### Carbonylated protein evaluation

Carbonylated proteins were evaluated in tissue homogenates as described previously [7]. Twenty µg of each spinal cord sample were denatured by 6% SDS and derivatized by 15-minute incubation with 2,4 dinitrophenyl hydrazine (DNPH; Sigma-Aldrich, Italy) at room temperature. Samples were separated on a 4–12% sodium dodecyl sulfate (SDS)-polyacrylamide gel by electrophoresis and transferred onto nitrocellulose membranes (Biorad, Italy). The membranes were incubated overnight with primary antibody specific versus DNPH (1∶5000; Sigma-Aldrich, Milan, Italy). Afterwards the procedure described for Western blotting analysis was followed. For each experiment the density of all bands showed in a lane was reported as mean. β-actin was used as loading control.

### Glial Fibrillary Acid Protein (GFAP) immuno reaction

Formalin-fixed cryostat sections (20 μm) were incubated for 1 h in blocking solution (Bio-Optica; Milan, Italy) at room temperature; and were then incubated for 24 h at 4°C in PBST containing rabbit primary antisera diluted 1∶1000 and 5% normal donkey serum. The primary antibody was directed against glial fibrillary acidic protein (GFAP; 1∶5000; Chemicon, Temecula, USA) for astrocyte staining. After rinsing in PBST, sections were incubated in donkey anti-rabbit IgG secondary antibody labeled with Alexa Fluor 488 (1∶1000, Invitrogen, Monza, Italy) at room temperature for 1 h. Negative control sections (no exposure to the primary antisera) were processed concurrently with the other sections for all immunohistochemical studies. We obtained a single optical density value for the dorsal horns by averaging the two sides in each rat, and these values were compared to the homologous average values from the vehicle-treated animals. Images were acquired by a motorized Leica DM6000B microscope equipped with a DFC350FX camera (Leica, Mannheim, Germany). Microglia and astrocyte morphology was assessed by inspection of at least three fields (40X 0.75NA objective) in the dorsal horn and cerebral areas *per* section. Quantitative analysis of GFAP -positive cells was performed by collecting at least three independent fields through a 20X 0.5NA objective. GFAP-positive cells were counted using the “cell counter” plugin of ImageJ. The GFAP signal in immunostained sections was quantified using FIJI software (distributed by ImageJ, NIH, Bethesda, Maryland, USA) by automatic thresholding images with the aid of the “Moments” algorithm, which we found to provide the most consistent pattern recognition across all acquired images. Area fraction (%) occupied by the thresholded GFAP signal revealed a common trend between GFAP expression and astrocyte cell number. Five spinal cord sections were analyzed for each animal.

### Determination of tissue platinum concentration

Plasma samples were pre-treated with nitric acid as described by [44] with minor modifications. Platinum levels were measured by Inductively Coupled Mass Spectrometry (ICP-MS) according to [45] in the “Laboratorio di Microanalisi” of the University of Florence.

### Statistical analysis

Results are expressed as mean ± SEM and analysis of variance (ANOVA) was performed. A Bonferroni’s significant difference procedure was used as post hoc comparison. All assessments were made by researchers blinded to cell or rat treatments. Slides from control and experimental groups were labeled with numbers so that the person performing the image analysis was blinded as to the experimental group. In addition all images were captured and analyzed by an investigator other than the one who performed measurements to avoid possible bias. Data were analyzed using the “Origin 8.1” software (OriginLab, Northampton, MA, USA).

## Results

### Primary rat astrocytes

**Figure 1:**
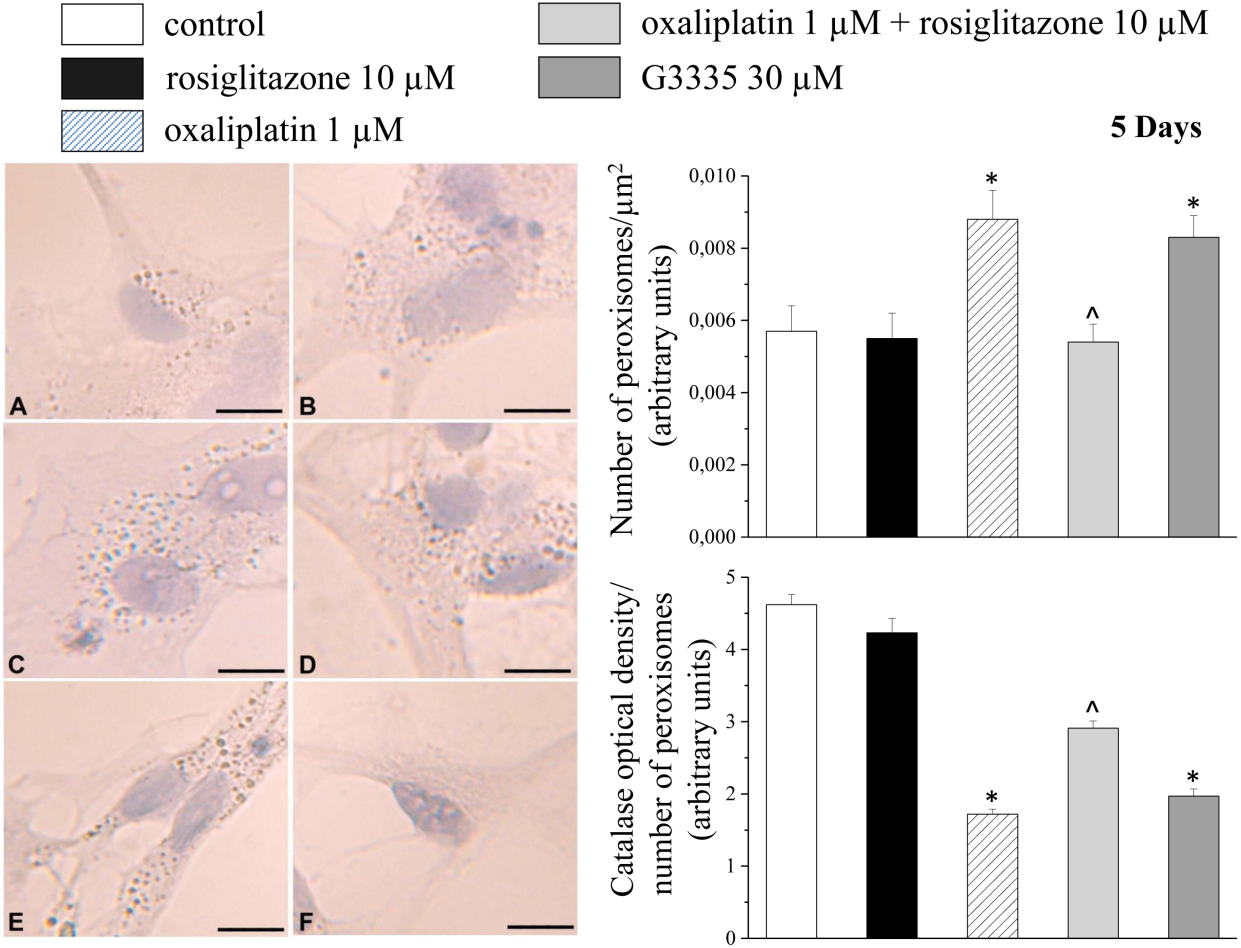
Catalase immunostaining in primary astrocytes. Cells (5·10^4^ cells/well) were incubated for 5 days with 10 µM rosiglitazone (B), 1 µM oxaliplatin (C), 1 µM oxaliplatin+10 µM rosiglitazone (D), 30 µM G3335 (E), negative control (F) in comparison to control condition (A). Representative images are shown in the left panel. Scale bar 50 µm. The measurements of the number of peroxisomes/µm^2^ and the catalase optical density per number of peroxisomes are shown in the upper and lower graphs, respectively. *P<0.01 vs control; ∧P<0.01 vs 1 µM oxaliplatin.

In astrocyte cell culture, peroxisomes were highlighted as catalase-positive organelles. After 5 day incubation, 1 µM oxaliplatin increased the number of peroxisomes by 54% (Figure 1). The PPARγ agonist rosiglitazone (10 µM) fully prevented the increase whereas 10 µM rosiglitazone *per se* (without oxaliplatin) did not modify peroxisome number. On the contrary the PPARγ antagonist G3335 (30 µM) was able to increase peroxisomes to the same extent of oxaliplatin (45%; Figure 1). G3335 effect was rosiglitazone-inhibitable (data not shown). The incubation for 48 h with 10 µM oxaliplatin induced similar effects (data not shown). Aimed to investigate the expression level of catalase in peroxisomes, we measured the ratio between the optical density value of catalase immunostaining and the number of peroxisomes. Oxaliplatin (1 µM, 5 day incubation) reduced the ratio by about 60% in comparison to the control value (Figure 1). G3335 (30 µM) decreased catalase expression similarly to oxaliplatin while 10 µM rosiglitazone significantly prevented alterations (Figure 1). After 48 h incubation, 10 µM oxaliplatin and 30 µM G3335 reduced the ratio by 26% and 49% (data not shown).

**Figure 2:**
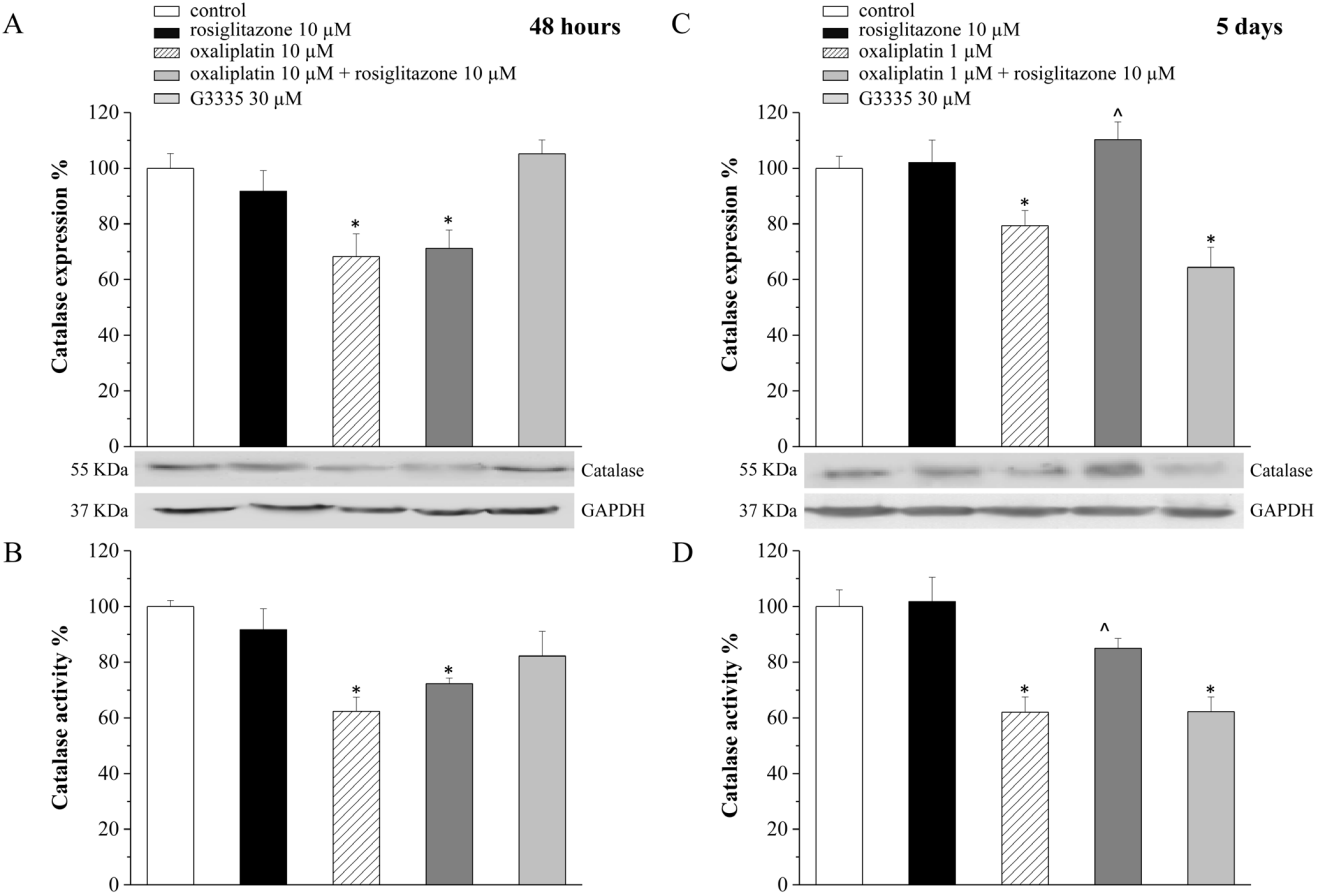
Expression and activity of catalase in astrocyte cell culture. Astrocytes (5·10^5^ cells/well) were treated with the PPARγ antagonist G3335 (30 µM) or with oxaliplatin (1 µM) in the absence or in the presence of the PPARγ agonist rosiglitazone (10 µM). Expression and activity were measured after 48 h- (A and B, respectively) or 5 day-treatment (C and D, respectively). GAPDH normalization was performed for each sample. Values are expressed as the mean ± S.E.M. percent of control of three experiments. Control condition was arbitrarily set as 100%. *P<0.05 vs control; ∧P<0.05 vs 1 µM oxaliplatin.

Similar alterations of catalase expression profile were measurable by Western blot. Protein level decreased from the control value of 100% to 68.3±8.1% after 48 h incubation with 10 µM oxaliplatin (Figure 2A) and to 79.4±5.4% after 5 day incubation with 1 µM oxaliplatin (Figure 2C). Moreover, the chemotherapeutic agent impaired the enzymatic activity of catalase. After 48 h, 10 µM oxaliplatin reduced catalase activity from 100% (control) to 62.3±5.1% (Figure 2B); five day incubation with 1 µM oxaliplatin reduced activity to 62.0±5.5% (Figure 2D). Effects evoked by oxaliplatin in 5 day incubation were mimicked by the PPAR-γ antagonist G3335 (30 µM). Catalase expression decreased to 64.4±7.2% (Figure 2C) and activity up to 62.2±5.3% (Figure 2D). The PPAR-γ agonist rosiglitazone (10 µM) prevented both oxaliplatin- (Figure 2D) and G3335-dependent (data not shown) alterations. Neither G3335 nor rosiglitazone were effective after 48 h of treatment. Aimed at evaluating the potential interaction between rosiglitazone treatment and the therapeutic property of oxaliplatin, we measured the viability of the human colon cancer cell line HT-29. Tables 1 and 2 show the lack of influence by the PPAR-γ agonist on the concentration-dependent (0.1–100 µM) oxaliplatin lethal effect after 48 h and 5 day incubation.

**Table 1:** HT-29 cell viability, 48 h.

	Oxaliplatin (µM)						
	0	1	3	10	30	100	300
**control**	100.0±2.4	99.7±1.1	95.1±1.1	89.1±1.4*	79.8±1.6*	38.6±3.7**	17.2±1.3**
**rosiglitazone 10 µM**	101.0±3.6	96.0±3.4	91.7±4.5	92.1±3.4	85.8±6.7*	44.9±4.3**	16.9±2.5**

Ht-29 cells were treated with increasing concentrations of oxaliplatin (1–300 µM) in the presence or in the absence of 10 µM rosiglitazone. Incubation was allowed for 48 h. Cell viability was measured by MTT assay. Control condition was arbitrarily set as 100% and values are expressed as the mean ± S.E.M. of three experiments. *P<0.05 and **P<0.01 in comparison to control (oxaliplatin 0 µM).

**Table 2:** HT-29 cell viability, 5 days.

	Oxaliplatin (µM)						
	0	0.1	0.3	1	3	10	30
**control**	100.0±4.5	98.0±5.3	91.8±2.4	92.6±3.2	90.9±3.4	88.1±2.3*	82.0±3.8*
**rosiglitazone 10 µM**	109.7±4.5	99.7±2.6	94.9±3.8	91.8±3.3	91.3±3.2	92.7±1.1*	75.4±4.8*

Ht-29 cells were treated with increasing concentrations of oxaliplatin (1–300 µM) in the presence or in the absence of 10 µM rosiglitazone. Incubation was allowed for 5 days. Cell viability was measured by MTT assay. Control condition was arbitrarily set as 100% and values are expressed as the mean ± S.E.M. of three experiments. *P<0.05 in comparison to control (oxaliplatin 0 µM).

### Behavioural measurements

**Figure 3:**
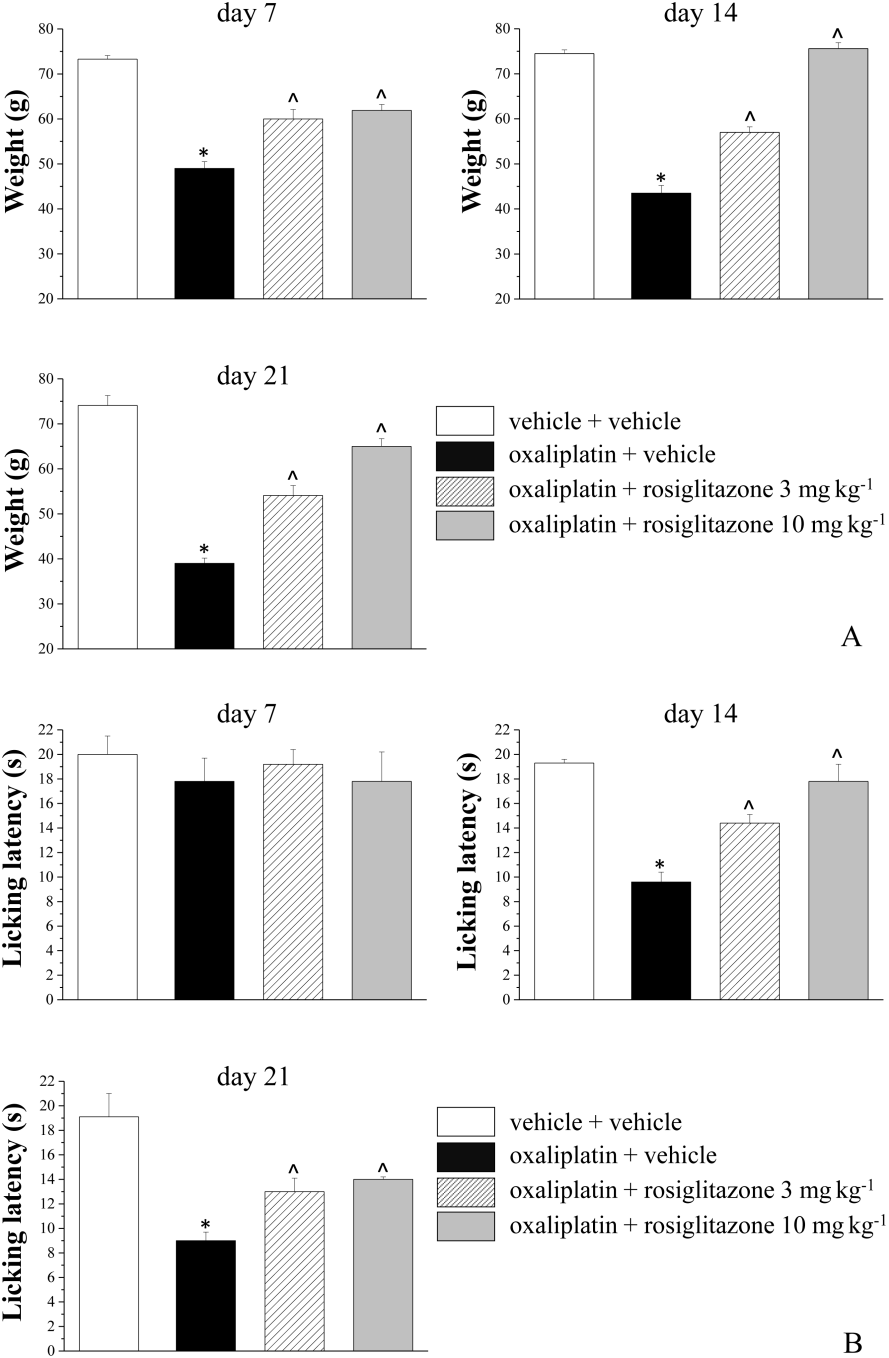
Pain threshold measurements. A) Noxious stimulus, Paw-pressure test. Rats were daily intraperitoneally treated with 2.4 mg kg^−1^ oxaliplatin (dissolved in 5% glucose). Rosiglitazone (3 and 10 mg kg^−1^, suspended in CMC) was per os daily administered starting from the first day of oxaliplatin administration. B) Non-noxious stimulus, Cold plate test. The response to a thermal stimulus was evaluated by cold plate test measuring the latency (seconds) to pain-related behaviors (lifting or licking of the paw). Control animals were treated with vehicles. Behavioral measures were performed on day 7, 14 and 21, 24 h after the last treatment. Each value represents the mean of 10 rats per group, performed in 2 different experimental set. *P<0.01 vs vehicle + vehicle (control); ∧P<0.01 vs oxaliplatin + vehicle.

**Figure 4:**
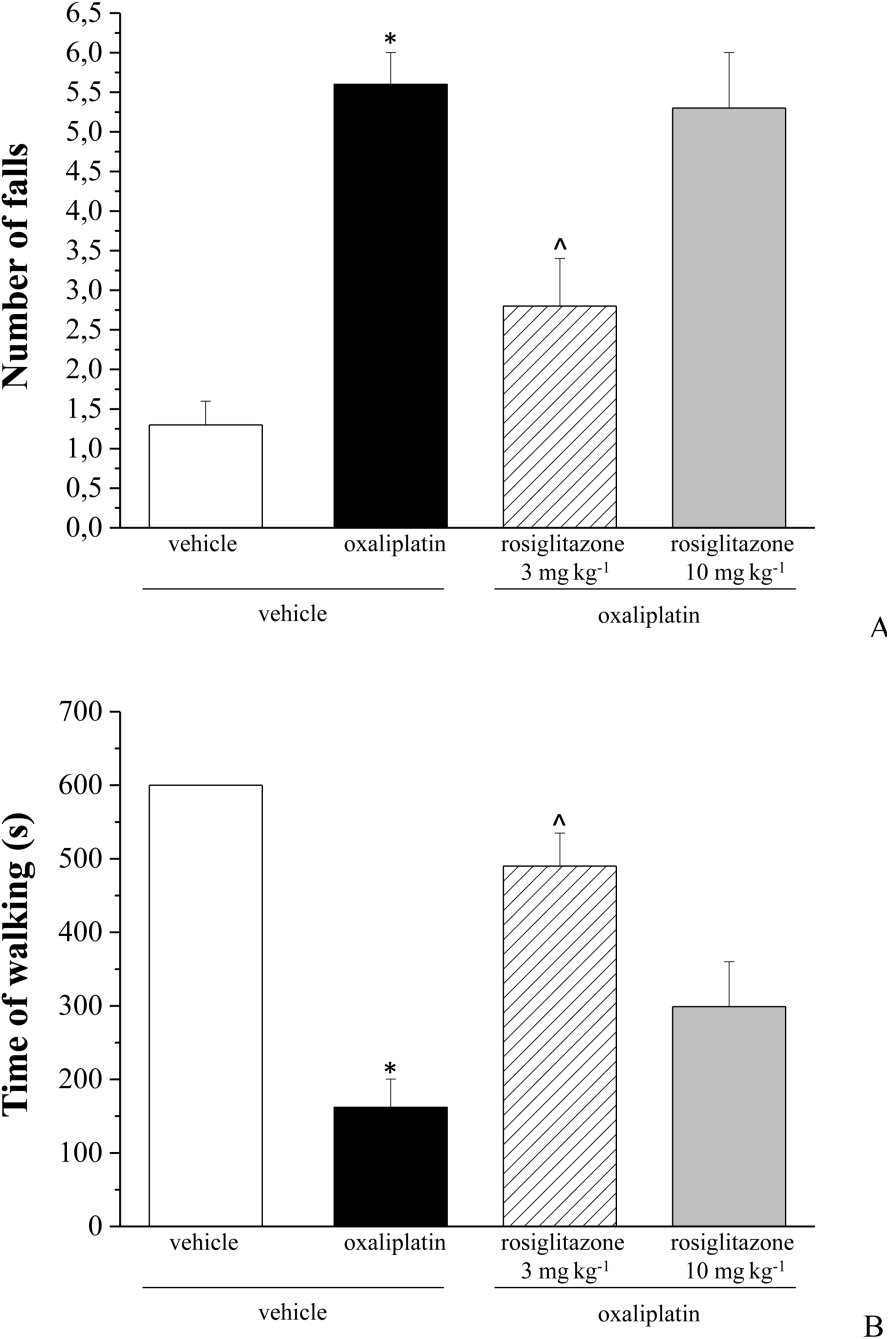
Motor coordination in oxaliplatin-treated rats. The integrity of the animals’ motor coordination was assessed using a rota-rod apparatus. Rats were placed on a rotating rod (10 rpm) for a maximum of 10 minutes (600 seconds). The number of falls (A) and the time spent in the balance (B) during 10 minutes were counted. Treatments (oxaliplatin 2.4 mg kg^−1^ i.p. and rosiglitazone 3 and 10 mg kg^−1^ p.o.) were performed daily. Motor coordination was evaluated on day 21, 24 h after the last treatment. Each value represents the mean of 10 rats per group, performed in 2 different experimental set. *P<0.01 vs vehicle + vehicle (control); ∧P<0.05 vs oxaliplatin + vehicle.

Seven days after the beginning of oxaliplatin treatment (2.4 mg kg^−1^ i.p., daily) pain sensitivity towards noxious stimulus (Paw pressure test) was altered. The weight tolerated on the posterior paw significantly decreased from the control value of 73.3±0.8 g to 49.0±1.5 g (Figure 3A). Rosiglitazone, 3 and 10 mg kg^−1^, per os administered daily, limited hypersensitivity increasing the tolerated weight (60.0±2.1 g and 61.9±2.3, respectively; Figure 3A). The progression of oxaliplatin-dependent neuropathic state on day 14 and 21 (Figure 3A) was reduced dose dependently by rosiglitazone (Figure 3A). In Figure 3B the withdrawal threshold to non-noxious thermal stimulus was shown. Cold plate test highlighted a decreased pain threshold starting from the 2^nd^ week of oxaliplatin treatment (9.6±0.8 s, oxaliplatin + vehicle group, in comparison to vehicle + vehicle group, 19.3±0.3 s). On day 14 pain threshold was increased by 49% and 84% in 3 and 10 mg kg^−1^ rosiglitazone-treated animals, respectively. Both rosiglitazone dosages were effective on day 21 increasing pain threshold by 39% and 49%, respectively (Figure 3B). On day 21, motor coordination was evaluated by Rota rod test measuring the walking time and the number of falls in 600 s. In comparison with control rats (number of falls 1.3±0.3, time 600 s) oxaliplatin treated animals fell down 5.6±0.4 times (Figure 4A) and maintained the balance for 162.0±38.5 seconds (Figure 4B) and. Three mg kg^−1^ rosiglitazone reduced the number of falls to 2.8±0.6 (Figure 4A) and improved the time of walking to 490±45 seconds (Figure 4B). The higher dosage of rosiglitazone was ineffective.

### *Ex vivo* evaluation

**Figure 5:**
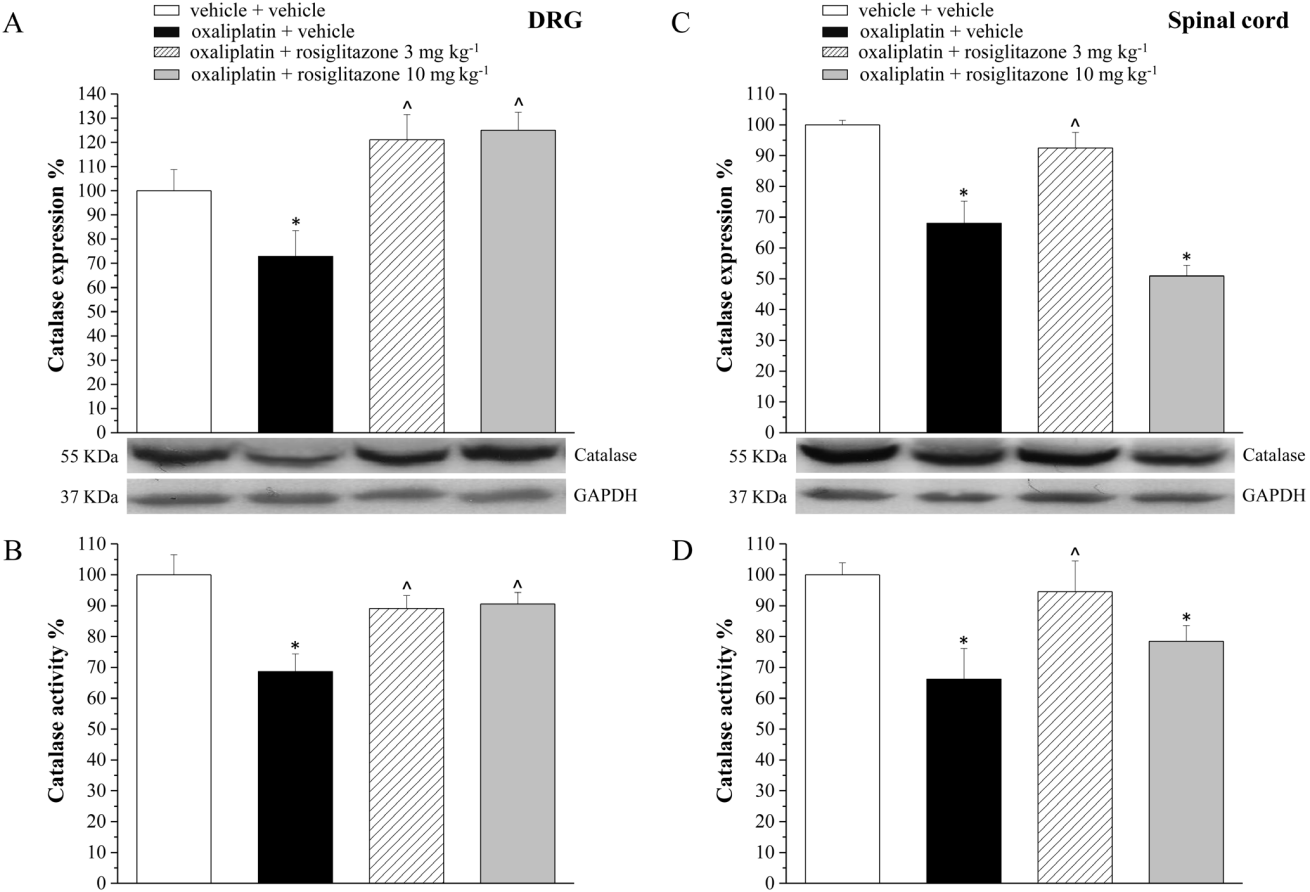
Expression and activity of catalase in the nervous tissue of oxaliplatin-treated animals. On day 21, dorsal root ganglia (DRG) and spinal cord were analyzed to measure both expression and activity of catalase. Densitometric analysis and representative Western blot of catalase expression in DRG (A) and spinal cord (C) are shown. GAPDH normalization was performed for each sample. Catalase enzymatic activity measurements in DRG (B) and spinal cord (D). Values are expressed as the mean ± S.E.M. percent of control of 10 rats per group, performed in 2 different experimental set. Each value represents the mean of *P<0.05 vs vehicle + vehicle; ∧P<0.05 vs oxaliplatin + vehicle.

**Figure 6:**
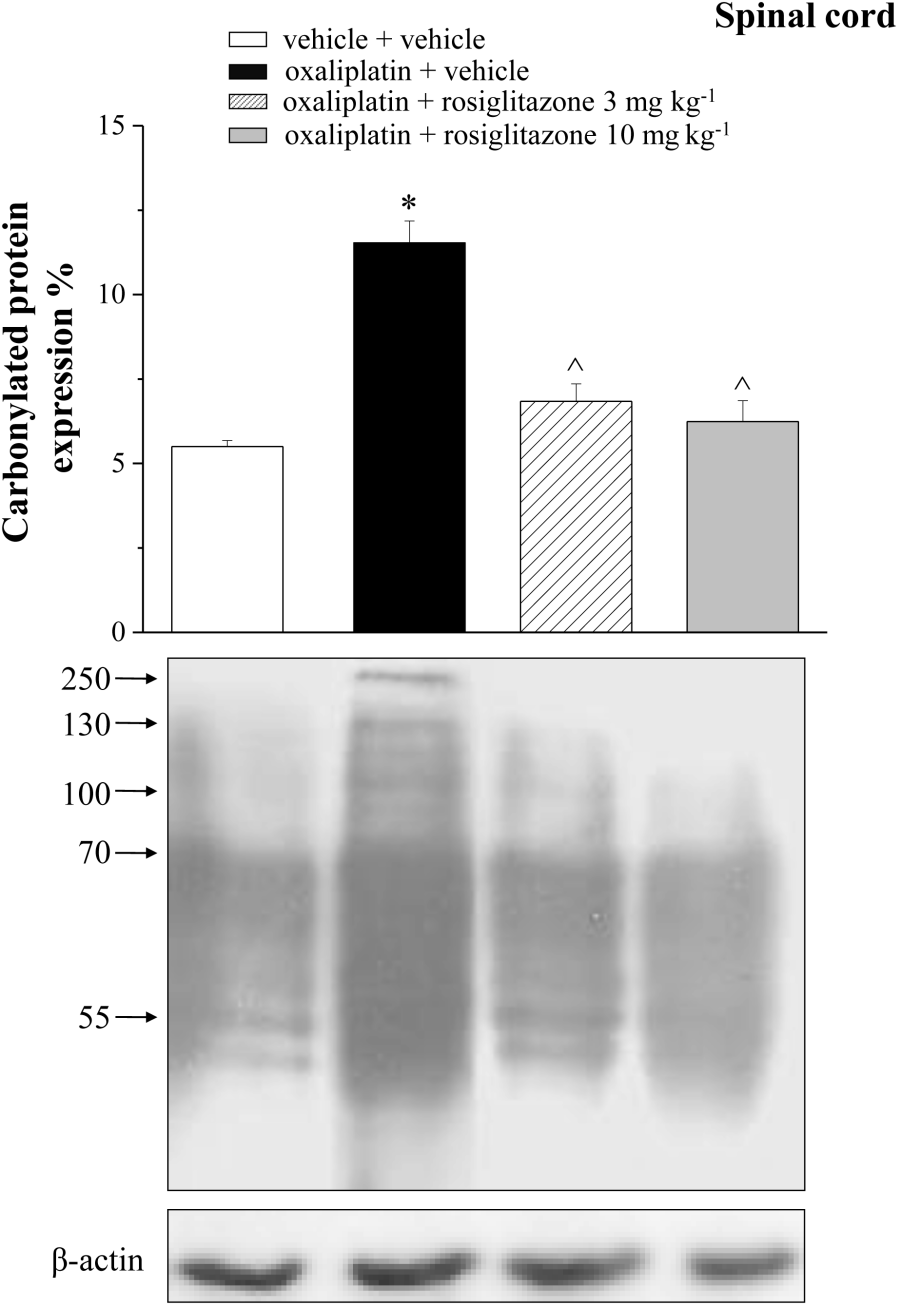
Levels of carbonylated proteins in the spinal cord of oxaliplatin-treated rats. At 21^th^ day, the lumbar tract of the spinal cord was explanted and analyzed to measure protein oxidation. Densitometric analysis (top panel) and representative Western blot (lower panel) are shown. β-actin normalization was performed for each sample. Values are expressed as the mean ± S.E.M. percent of control of 10 rats per group, performed in 2 different experimental set. Each value represents the mean of *P<0.05 vs vehicle + vehicle; ∧P<0.05 vs oxaliplatin + vehicle.

**Figure 7:**
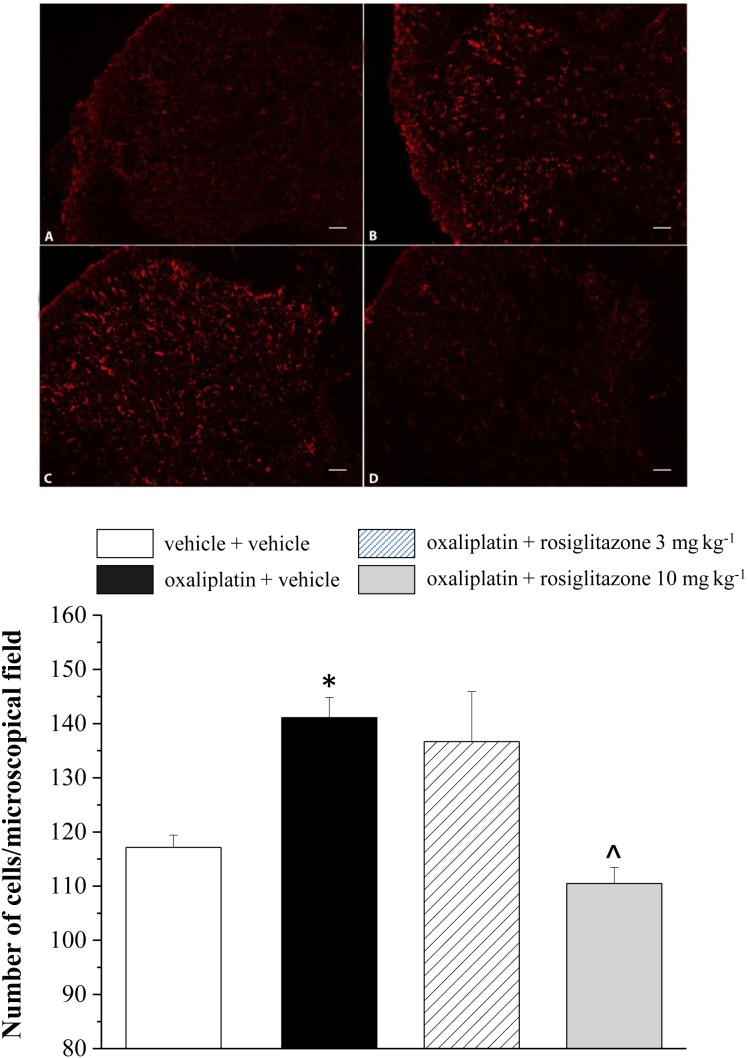
Glial profile in spinal cord scored with GFAP-positive cells in the dorsal horn of the lumbar tract. Transverse sections of spinal cord imaged with 20X objective of A) vehicle + vehicle, B) oxaliplatin + vehicle, C) and D) oxaliplatin + rosiglitazone 3 and 10 mg kg^−1^, respectively. Scale bar 50 µm. In the lower panel quantitative analysis of cellular density is shown. Each value represents the mean ± S.E.M. of 10 rats per group, performed in 2 different experimental sets. *P<0.05 vs vehicle + vehicle; ∧P<0.05 vs oxaliplatin + vehicle.

Aimed to evaluate in vivo the relationship between oxaliplatin neurotoxicity and peroxisome, catalase expression and activity were measured in peripheral and central nervous tissue on day 21 of anticancer treatment. In DRG, catalase expression decreased up to 72.9±10.7% in comparison to the control value (100±8.7%, Figure 5A), the enzymatic activity was reduced up to 75.6±5.8% (Figure 5B). Rosiglitazone (3 mg kg^−1^ and 10 mg kg^−1^) prevented catalase impairment (Figure 5A and B). Figures 5C and 5D show the oxaliplatin-induced decrease of catalase expression (68.0±7.2%) and functionality (66.2±9.9%) in the spinal cord. The lower dosage of rosiglitazone (3 mg kg^−1^) only, was able to prevent catalase alteration in spinal cord (Figure 5C and D). Moreover, both the doses of rosiglitazone limited the oxidative damage induced by oxaliplatin. As shown in Table 3, the lipid peroxidation promoted by the anticancer agent (up to 4 times as the basal value) was prevented by 3 and 10 mg kg^−1^ rosiglitazone. Protein oxidation, evaluated as increase of the expression level of carbonylated protein was significantly prevented by rosiglitazone (Figure 6). Aimed to better understand the role of astrocyte cells in the modulation of neuropathic pain, we performed immunohistochemical analysis of GFAP-positive cells in the dorsal horn of the spinal cord (Figure 7). The oxaliplatin-dependent increase of cell number (about 25% increase in oxaliplatin + vehicle as compared to vehicle + vehicle), was prevented by 10 mg kg^−1^ rosiglitazone (Figure 7).

**Table 3:** Lipid peroxidation in spinal cord.

	TBARS
	(μmol/mg protein)
**vehicle + vehicle**	35.05±7.25
**oxaliplatin + vehicle**	133.02±23.45*
**oxaliplatin + rosiglitazone 3 mg kg^−1^**	29.68±7.12∧
**oxaliplatin + rosiglitazone 10 mg kg^−1^**	39.27±93.45∧

On day 21, the lumbar tract of the spinal cord was explanted for the analysis of lipid peroxidation. Data were expressed as mean ± SEM of Thiobarbituric Acid Reactive Substances (TBARS) levels (µmol/mg protein). Each value represents the mean of 10 rats per group, performed in 2 different experimental set. *P<0.01 vs vehicle + vehicle (control); ∧P<0.05 vs oxaliplatin + vehicle.

## Discussion

Neuropathy is a dose limiting side effect of many anticancer agents, including oxaliplatin. In the clinical practice, the common human dosage of oxaliplatin is 85 mg/m^2^ and cumulative doses higher than 1000 mg/m^2^ [4], [5] causes chronic neuropathy in approximately 50% of patients. The human plasmatic concentration of inorganic platinum after a single i.v. injection of 85 mg/m^2^ is on average about 3 µg/mL and only limited accumulation is observed in plasma after repeated cycles (five consecutive cycles at 85 or 130 mg/m^2^ every 3 weeks) [46], [47].

The model used for the present research is consistent with the clinical practice. 2.4 mg kg^−1^ oxaliplatin corresponds to the common human dosage (considering the Km factor 37 for the conversion of animal doses to the Human Equivalent Dose [48], [49]). The daily repeated administration of 2.4 mg kg^−1^ performed in the animal model allows to obtain a cumulative dose of 36 mg kg^−1^ corresponding to 1332 mg/m^2^. This dosage mimics the clinical cumulative oxaliplatin dose causing chronic neuropathy. Moreover, in our condition the inorganic platinum plasmatic levels after 21 days of treatment is 3.573±0.217 µg/mL in line to human plasma concentration.

Among the debated biomolecular mechanisms of nervous damage induced by platinum derivatives [50], we previously indicated the oxidative stress as exploitable pathological target for the treatment of chemotherapy-induced neuropathy. In our model, a relationship between oxaliplatin-induced neuropathic pain and oxidative damage was shown [7]. The cellular redox balance is mainly regulated by two organelles, mitochondria and peroxisomes, and the oxidative homeostasis is due to their cooperation [51]. Mitochondrion was extensively studied and it is considered a pivotal target of platinum neurotoxicity since Zheng et al. [10] described an increase in the incidence of swollen and vacuolated mitochondria in peripheral nerve axons in animals treated with oxaliplatin. Moreover, we have recently confirmed that oxaliplatin promotes a significant cytosolic release of cytochrome C in astrocyte cell culture, indicating a mitochondrial suffering [52]. On the contrary, the peroxisomal compartment was poor analyzed and its relevance in cellular redox metabolism has been underestimated for a long time [22]. Peroxisomes are single-membrane bound organelles with a protein-rich matrix and play a key role in both the production and scavenging of ROS in the cell [15]. Catalase is the most important antioxidant defense enzyme in mammalian peroxisomes breaking down hydrogen peroxide to water and oxygen [26], [53], [54]. Catalase alterations are highlighted in many neurodegenerative conditions correlate to oxidative damage. Brain of aged patients showed a reduced catalase functionality [55], similarly catalase activity was affected in a rat model of Parkinson’s disease [56]. Catalase impairment was also described in neuropathic conditions since decreased catalase efficiency was described in sciatic nerve of rats affected by diabetic neuropathy [57] as well as in brain regions of animals with delayed neuropathy induced by organophosphate [58]. On the other hand, the oxidative unbalance of these painful conditions has never been associated with peroxisome alterations.

The present results highlight the relevance of peroxisomes in oxaliplatin-dependent neurotoxicity. In astrocyte culture, glial cells implied in the development and maintenance of chronic pain [59], [60] and sensitive to the platinum drug toxicity [8], oxaliplatin increases the number of peroxisomes. Peroxisomes are able to respond to physiological changes in cellular environment adapting their number, morphology, enzyme content and metabolic functions [61]. In particular, alterations of peroxisome number were described during carcinogenesis and liver cirrhosis, suggesting proliferative mechanisms as well as peroxisome division [61]. In our condition, the increase in peroxisome number is insufficient to provide the physiological level of catalase functionality. Both activity and expression of this antioxidant enzyme are reduced in astrocytes by oxaliplatin treatment. Astrocytes are generally less susceptible to oxidative injury than neurons and provide for their health and functionality [62], [63]. The dysregulation of astrocyte antioxidant machinery can influence neuron functionality and evoke nervous circuit alterations. Accordingly, in the rat model of oxaliplatin-induced neuropathy a similar alteration of catalase is highlighted in DRGs and spinal cord. These data suggest an impairment of peroxisome that may participate to oxaliplatin-induced redox unbalance previously observed in astrocyte culture as well as in the nervous tissue of neuropathic animals [7], [8].

Oxaliplatin-induced alteration of catalase, in terms of activity and expression, is comparable to that evoked by the pharmacological blockade of PPARγ (by the selective and reversible PPARγ antagonist G3335 [41]). PPARs belong to a nuclear receptor superfamily actively involved in immunoregulation. Membrane lipid composition, cell proliferation, sensitivity to apoptosis, energy homeostasis, and various inflammatory transcription factors are regulated by the trans-repression capabilities of these receptors [64]. The γ subtype of PPARs is expressed both in neurons [65] and glia cells [66] and PPARγ stimulation protects neuronal and axonal damage induced by oxidative stimuli [67]. This property has been associated with a concomitant increase in the enzymatic activity of catalase [67] accordingly to the evidence of a direct modulation of this enzyme by PPARγ [25]. The similarity of oxaliplatin- and G3335-mediated effects on astrocyte catalase and peroxisome number suggests a common dysregulation of these organelles. Since oxaliplatin impairs catalase in 48 h whereas G3335 needs 5 days, we can hypothesize a direct effect of oxaliplatin on the peroxisome machinery. On the other hand, 5 days incubation with the selective PPARγ agonist rosiglitazone, reduces the enzymatic failure promoted by both oxaliplatin and G3335 and normalizes the peroxisome number. Accordingly, the repeated administration of rosiglitazone improves catalase efficiency in the nervous tissue of oxaliplatin-treated rats and prevents spinal oxidative alterations reducing the lipid peroxidation and carbonylated protein levels. The maintenance of the defensive properties of catalase, and the consequent redox balance improvement, are concomitant with the control of pain exerted by the PPARγ agonist. A relationship between pain and catalase impairment is suggested. Rosiglitazone reduces oxaliplatin-dependent alterations of the pain threshold when both noxious or non-noxious stimuli are used. The anti-neuropathic effect is dose- and time-dependent till day 14. On day 21, the effect of 3 and 10 mg kg^−1^ is similar in the Cold plate test. On the same day, the low dose treated animals (3 mg kg^−1^) show an improvement in motor coordination and a significant restoration of catalase expression and activity in the central nervous system, whereas the beneficial effect of the higher dose (10 mg kg^−1^) disappears. These evidences suggest the need of a mild PPARγ stimulation to obtain a protective antineuropathic effect. Interestingly, the 10 mg kg^−1^ dosage prevents the increase of astrocyte number in the spinal cord, on the contrary the lower dose is ineffective. Glia cells contribute to the persistence of pain [68] as well as to several omeostatic functions above all neuroprotection [69]. The block of glial-related signals impairs functional recovery after nerve injury [70], suggesting that *tout court* glial inhibition may relieve pain but hinders the rescue mechanisms that protect nervous tissue. Accordingly, the present data suggest that the lower dose of rosiglitazone (which is unable to decrease astrocyte cell number) yields the better balance between neuroprotective and anti-hyperalgesic effects. On the other hand, the effect of rosiglitazone on glia cells is not univocally depending by pathological condition and by CNS area. In rats, 0.1 mg kg^−1^ rosiglitazone (i.p.) was able to decrease the cognitive impairment after status epilepticus and to inhibit astrocyte activation in the striatum [71]. On the contrary, in a rat Parkinson’s model, the neuroprotective effect induced by 3 mg kg^−1^ rosiglitazone (i.p.) is concomitant with an increase in GFAP expression in the hippocampus [72]. Noteworthy, the capability of rosiglitazone to penetrate the blood brain barrier is debated [73]–[75], though central effects have been demonstrated [76].

Finally, it is important to highlight the absence of interaction between the PPAR-γ agonist and the lethal effect exerted by oxaliplatin on the human colon cancer cells HT-29. Moreover, thiazolidinediones reduce the growth of different tumors, arresting cancer cell proliferation by affecting cell cycle checkpoints or inhibiting growth factors [77]. Preclinical and clinical studies have demonstrated the antitumoral effect of rosiglitazone alone or in combination [78].

In summary, oxaliplatin-dependent neurotoxicity alters peroxisome functionality. The PPARγ agonist rosiglitazone prevents these phenomena and controls pain. The optimal profile shown by the lower dosage suggests the mild stimulation of PPARγ as possible approach to the oxaliplatin neuropathy.
